# Calcium Bioavailability of *Opuntia ficus-indica* Cladodes in an Ovariectomized Rat Model of Postmenopausal Bone Loss

**DOI:** 10.3390/nu12051431

**Published:** 2020-05-15

**Authors:** Michelle Quintero-García, Elsa Gutiérrez-Cortez, Alejandra Rojas-Molina, Monsserrat Mendoza-Ávila, Alicia Del Real, Efraín Rubio, Daniel Jiménez-Mendoza, Isela Rojas-Molina

**Affiliations:** 1 Programa de Maestría en Ciencias Químico Biológicas, Facultad de Química, Universidad Autónoma de Querétaro, Cerro de las Campanas S/N, Querétaro C.P. 76010, Mexico; adr_mich@hotmail.com; 2 Laboratorio de Química Medicinal, Facultad de Química, Universidad Autónoma de Querétaro, Cerro de las Campanas S/N, Querétaro C.P. 76010, Mexico; rojasa@uaq.mx; 3 Laboratorio de procesos de transformación y tecnologías emergentes en alimentos, Facultad de Estudios Superiores-Cuautitlán, Universidad Nacional Autónoma de México, Km 2.5 Carretera Cuautitlán–Teoloyucan, San Sebastián Xhala, Cuautitlán-Izcalli C.P. 54714, Mexico; elsaneqpm@yahoo.com.mx; 4 Programa de Maestría en Ciencias de la Nutrición Humana, Facultad de Ciencias Naturales, Universidad Autónoma de Querétaro, Av. de las Ciencias S/N, Juriquilla C.P. 76230, Querétaro, Mexico; monsse_ph@hotmail.com; 5 Centro de Física Aplicada y Tecnología Avanzada, Universidad Nacional Autónoma de México, Juriquilla C.P. 7600, Querétaro, Mexico; adelreal@unam.mx; 6 Centro Universitario de Vinculación y Transferencia de Tecnología, Benemérita Universidad Autónoma de Puebla, Centro Universitario, Col. San Manuel S/N, Puebla C.P. 72540, Mexico; efrainrubio@yahoo.com; 7 Departamento de Ingeniería Física, División de Ciencias e Ingenierías, Universidad de Guanajuato, Campus León, Lomas del Bosque 103, Col. Lomas del Campestre, León C.P. 37150, Guanajuato, Mexico; jmd_pepe@hotmail.com; 8 Departamento de Ingeniería Electromecánica, Tecnológico Nacional de México/ITS de Purísima del Rincón. Blvd. Del Valle 2301, Col. Guardarrayas, Purísima del Rincón C.P. 36413, Guanajuato, Mexico

**Keywords:** *Opuntia ficus-indica*, calcium bioavailability, bone mineral density, remodeling biomarkers, ovariectomy, osteoporosis

## Abstract

Osteoporosis is a disease of the skeletal system characterized by low bone mass and bone weakening, which increase the risk of fracture. This disease is associated with menopause because hypoestrogenism induces the maturation and activation of osteoclasts. In addition, a low dietary intake of calcium leads to low bone mineral density and postmenopausal osteoporosis. The objectives of this work were to determine calcium bioavailability of *Opuntia ficus-indica* cladodes at a late maturity stage and to assess its contribution in improving bone health in an ovariectomized rat model. Two-month-old Wistar female rats (*n* = 35) were used and distributed in seven experimental groups: (i) control group (Crtl), (ii) sham group (SH), (iii) ovariectomized group (OVX), (iv) ovariectomized group supplemented with calcium citrate (CCa), (v) ovariectomized group supplemented with *O. ficus-indica* powder (NI), (vi) ovariectomized group supplemented with soluble fiber from *O. ficus-indica* (FS) and (vii) ovariectomized group supplemented with insoluble fiber from *O. ficus-indica* (FI). Our results showed that calcium in the soluble fiber of *O. ficus-indica* is bioavailable and contributes to improve the physical, densitometric, biomechanical and microstructural properties of bones in ovariectomized rats. These findings indicated that *O. ficus-indica* cladodes at a late maturity stage represent a good source of bioavailable calcium and consumption of these cladodes might be beneficial for the prevention of osteoporosis and other bone diseases.

## 1. Introduction

An adequate bone mineralization is achieved through calcium and vitamin D diet ingestion. Foods that have high calcium content are dairy products; however, people with lactose intolerance are predisposed to low calcium intake, and therefore are at higher risk of bone demineralization [1]. The recommended daily intake (RDI) for calcium is 1200 mg/day in adults. The deficiency of this mineral in diet is directly related to diseases, such as osteoporosis, as well as to the propensity to suffer bone fractures [2]. In Mexico, it has been estimated that approximately 168 women and 98 men per 100,000 people have a proximal femur fracture, which means that one in 12 women and one in 20 men over 50 years of age will suffer a fracture [3]. All bone cells (osteoblasts, osteoclasts and osteocytes) contain functional estrogen receptors (ERs), which play an important role in bone metabolism and inhibition of osteoclast differentiation. Moreover, ER activation promotes osteoclast apoptosis, subsequently reducing bone resorption [4]. Currently, there are several treatments for the prevention of osteoporosis, among which, dietary supplements that contain calcium salts, such as calcium carbonate and citrate, are widely employed. Unfortunately, these supplements cause some gastrointestinal complications [5]. Vitamin D supplementation is also used in the preventive treatment of osteoporosis, however, its complete absorption is very limited in geriatric patients, since they barely carry out any type of exercise, such as walking, to take advantage of UV rays to achieve vitamin D fixation [6]. Another prevention and treatment option for osteoporosis is hormone replacement, which induces antiresorptive effects and has been used for decades in menopausal and postmenopausal women; nevertheless, it is accompanied by side effects associated with increased risk of breast cancer [7]. Our research group previously reported that *Opuntia ficus-indica* (nopal) has high levels of calcium, approximately 164 mg/100 g dried weight [8]. We also found that calcium content in *O. ficus-indica* increases approximately by 70% in cladodes at late maturity stages compared with that of cladodes at an early stage [9]. Moreover, we demonstrated that calcium content in the soluble fiber of *O. ficus-indica* cladodes is significantly higher than that found in the insoluble fiber. Interestingly, most of this mineral in the soluble fiber is in the form of calcium carbonate, whereas it is in the form of calcium oxalate in the insoluble fiber [10]. To our knowledge, there are no studies regarding the effects of calcium present in *O. ficus-indica* cladodes and fibers extracted from this cactus to improve bone properties in ovariectomized rats. Based on the aforementioned data, we hypothesized that calcium in *O. ficus-indica* cladodes is bioavailable and useful to enhance bone health. Therefore, the objectives of this work were to determine the bioavailability of calcium in *O. ficus-indica* cladodes at a late maturity stage and to assess its contribution to improve physical, densitometric, biomechanical, microstructural and mineral content properties of bones in an ovariectomized rat model of postmenopausal bone loss.

## 2. Materials and Methods

### 2.1. Vegetal Material

*Opuntia ficus-indica* cladodes were harvested during the spring of 2016 in the experimental field of Amazcala in the Engineering Department of the Autonomous University of Queretaro. The cladodes were collected at 135 days of maturity stage from the sprout.

### 2.2. Preparation of O. ficus-indica Powder

The thorns of cladodes were removed, and then the cladodes were cut and placed in stainless steel trays, which were introduced in a forced air oven (BG Didacta, Torino, Italy) to be dehydrated at 70 °C for 12 h [11]. Subsequently, the cladodes were ground in a hammer mill (Pulvex 200, Mexico).

### 2.3. Extraction of Soluble and Insoluble Fibers from O. ficus-indica Cladodes

Suspensions of *O. ficus-indica* powder were prepared for the extraction of soluble and insoluble fibers from the cactus based on the methodology reported by Rojas-Molina et al. [10].

### 2.4. Determination of Calcium and Phosphorus Content in O. ficus-indica Cladodes

Analyses of calcium (Ca) of dehydrated *O. ficus-indica* cladodes were carried out in triplicate by using a double-beam atomic absorption spectrophotometer (Analyst 300, Perkin Elmer, Boston, MA, USA). Quantifications were performed in accordance with the methods established by the Association of Official Analytical Chemists (AOAC, 2000) [12]. Phosphorus content was analyzed according to previous reports [9].

### 2.5. Experimental Design

Thirty-five adult female Wistar rats (2 months of age), which underwent ovariectomy surgery, were used as an experimental model of postmenopausal osteoporosis. The rats were randomly assigned to the experimental groups. Experimental subjects were placed in stainless steel metabolic cages under controlled conditions of temperature and light (light–dark cycles of 12 h:12 h). The study was approved by the Bioethics Committee of the Natural Sciences Department of the Autonomous University of Queretaro. Animals had ad libitum access to deionized water and diets. The subjects were randomly classified into 7 experimental groups with 5 rats in each one as follows: (i) control group fed with diet AIN-93M for maintenance of adult rodents (Ctrl group), (ii) sham group fed with diet AIN-93M (SH group), (iii) group of ovariectomized rats fed with diet AIN-93M (OVX group), (iv) ovariectomized group fed with diet AIN-93M adjusted with calcium contained in the soluble fiber extracted from *O. ficus-indica* cladodes (FS group), (v) ovariectomized group fed with AIN-93M diet adjusted with calcium contained in the insoluble fiber extracted from *O. ficus-indica* cladodes (FI group), (vi) ovariectomized group fed with diet AIN-93M adjusted with calcium contained in *O. ficus-indica* cladodes (NI group) and (vii) ovariectomized group fed with diet AIN-93M adjusted with calcium from calcium citrate (Cca group). The experimental period was 9 weeks. The experimental diets were prepared with AIN-93M modifications (Table 1) including an addition of vitamin mix (AIN-93-VX, Harlan Inc., IN, USA, TD 94047) and mineral mix without calcium (AIN-93-MX, Harlan Inc., IN, USA, TD 04374). The calcium content in all diets was 5 g/kg diet and the calcium source in the control diet was calcium carbonate (Merck 2066, Darmstadt, Germany). Different amounts of dehydrated *O. ficus-indica* cladodes and insoluble and soluble dietary fiber extracted from *O. ficus-indica* cladodes were added to experimental diets as the calcium source to achieve the aforementioned calcium content. Energy values were calculated with standard factors as follows: 4 kcal for available carbohydrates and proteins and 9 kcal for lipids.

### 2.6. Chemical Composition of Experimental Diets

Moisture, ashes, total lipids and crude protein (*N* × 6.25) were measured in all diets as shown in Table 2. The total carbohydrate content was analyzed by difference using the formula: % carbohydrates = 100 – (% moisture + % proteins + % lipids + % minerals + % crude fiber) [14]. The quantification of calcium (Ca), phosphorus (P), magnesium (Mg) and potassium (K) in the diets was carried out by inductively coupled plasma mass spectrometry (ICP-MS) and using a ICP-EOS equipment (Variant 730-ES, Kyoto, Japan). All tests were performed following the official methods [12].

### 2.7. Evaluation of Serum Estrogen Levels

In order to corroborate the success of the surgical removal of ovaries, blood was collected from the caudal vein of the rats one day before starting the treatment and at the end of the experiment. Serum separation was performed by centrifugation at 3000 rpm (LXJ-802, Thermo Scientific, PA, USA) and estrogen levels were measured according to the previous reports of Choi et al. [15] by an immunoassay and by using a commercial kit (ELISA Kit, Cat. No. 582251-96S, Cayman Chemical Company, MI, USA).

### 2.8. Calcium Bioavailability

Calcium bioavailability was determined through the apparent calcium absorption and calcium balance according to the Equations (1) and (2) [16]:

(1) $$\mathrm{Apparent}\;\mathrm{calcium}\;\mathrm{absorption}\;\left(\%\right)=\frac{\mathrm{calcium}\;\mathrm{intake}-\mathrm{calcium}\;\mathrm{excretion}\;\;}{\mathrm{calcium}\;\mathrm{intake}}\times 100\;\;\;\;\;\;$$

(2) Calcium balance (%)= Calcium intake−(urinary calcium+calcium in feces)      

The amount of food consumption was measured for four continuous days before the animals were sacrificed. Ingested calcium, fecal calcium and urine calcium were measured [17].

### 2.9. Assessment of Femoral Dimensions and Weight

Experimental animals were fasted for 12 h before sacrifice by decapitation. Afterward, the right and left femurs were excised, and adherent soft tissue and bone marrow were removed. Bones were stored at 4 °C until further analysis. Weight gain was measured, as well as the length and diameter of the bones, according to a method described previously in [9]. Briefly, the fresh femurs were weighed and measured using a Vernier caliper (Absolute Digimatic, Mitutoyo, Japan). Length measurements were made from the greater trochanter to the lateral condyle. The width and thickness were measured at the midpoint (diaphysis) of the femurs. Subsequently, the femurs were labeled and stored at −20 °C until analysis.

### 2.10. Analyses of the Biomechanical Properties of Femurs

Biomechanical properties of femoral bone were determined in the right femur through the force required to break the bone by using a material testing machine (Mod. Z005, load cell 5000N, Zwick/Roell, Ulm, Germany), employing the TestXpert Intelligent testing version 12.0 software [9]. In brief, the samples were defrosted and all the measurements were carried out at room temperature. The failure load of femurs was evaluated by three-point bending (maximum breaking force of failure when the load is applied in a perpendicular plane to the longitudinal axis of the femur, denoted by the symbol P_max_) and compression tests (maximum force of failure when the force is applied in a vertical plane to the longitudinal axis of the femur, denoted by the symbol F_max_). Additionally, Young’s modulus or elastic modulus (*E*) was calculated. All the tests were evaluated in the mid-diaphyseal region of the femur.

### 2.11. Determination of Calcium and Phosphorus Content in the Femurs

Left femurs were used for Ca and P analysis. Both minerals were determined by inductively coupled plasma mass spectrometry (ICP-MS) by using an ICP-EOS equipment (730-ES, Agilent, Santa Clara, CA, USA) [9]. First, the bones were defrosted and crushed with a laboratory mortar and then oven-dried at 60 °C to a constant weight. The dry samples were weighed and a mixture of nitric acid and perchloric acid at a ratio of 3:1 was added to digest the samples (MarsXpress, CEM, Matthews, NC). Then, the samples were transferred and diluted with 10 mL of distilled water. Reagent blanks were prepared with the same digestion procedures.

### 2.12. Analyses of Bone Mineral Density

Rats belonging to the experimental groups were subjected to densitometry at the beginning and at the end of the experiment by using a simple X-ray equipment (X-Mind^®^, Satelec, Cologne, France) with a potential of 70 kV, a current of 8 mA and a wavelength of 0.177 × 10^−10^ m (DG-073B-DC X-ray tube, Toshiba, Tokyo, Japan), as well as a detector (S10835 CMOS sensor, Hamamatsu, Iwata, Japan) and a computer platform, in order to determine bone mineral density (BMD) [9,18]. The neck and diaphysis of femurs (cortical tissue) and metaphysis (cortical-trabecular tissues) were selected for measurements. For each bone, three measurements were obtained.

### 2.13. Evaluation of the Microstructural Properties of Rat Femoral Bone

Microstructural parameters of the femurs, such as trabecular separation (Tb.Sp), trabecular thickness (Tb.Th) and cortical thickness (Ct.Wi), were determined in a scanning electron microscope (JSM 6060LV, Jeol, Japan) as previously described [9,19]. Prior to the analysis, the left femurs were exposed at 130 °C in a Papin reactor (6 L, Cinsa, Mexico City, Mexico) for 1 h, and thereafter, the bones were dried in an oven (FE-295A, Felisa, Zapopan, Jalisco, Mexico) at 60 °C to a constant weight. Subsequently, the femurs were each cut longitudinally from the intercondylar line to the diaphysis with a disc diamond saw (Diaflex-Transvident 350-352, Berlin, Germany). The femur sections were incubated with protease (P-6911, 1% *w*/*v*, Sigma Chemical Co., St. Louis, MO, USA) at 37 °C for 24 h. Bones were subjected to a second digestion process with aminopeptidase (P-6887, 0.4% *w*/*v*, Sigma Chemical Co.) at 37 °C for 18 h. Then, lipids in bones were removed with ethyl ether (Cat. No. 9240-03, J.T. Baker Center Valley, PA, USA) and acetone (Cat. No. 9006-03, J.T. Baker Center Valley, PA, USA) on a shaker (AR-100, Daigger, Vernon Hills, IL, USA) for 12 h. The femurs were dried at 60 °C to a constant weight. Finally, the bones were mounted on stubs and coated with gold using an ion sputter for observation.

### 2.14. Measurement of Bone Remodeling Biochemical Biomarkers

At the beginning of the biological experiment, blood samples were collected from the caudal vein of each rat and serum separation was performed. Serum samples were stored at 4 °C for later analysis. At the end of the study, animals were sacrificed by decapitation, and their serum was obtained. Serum levels of three bone formation biomarkers, including osteocalcin (ELISA Kit Bioassay, Cat. No. 200767-96T, USB Biologicals, MA, USA), amino-terminal procollagen type 1 (ELISA Kit Bioassay, Cat. No. 202110-96T USB Biologicals, MA, USA) and alkaline phosphatase (ELISA Kit Bioassay, Cat. No. 183576-96T, USB Biologicals, MA, USA), were determined according to the methodology reported by Lee [20].

### 2.15. Statistical Analyses

Results are expressed as mean values ± standard deviation (SD). All data were analyzed using one-way analysis of variance (ANOVA) followed by Tukey’s test with α = 0.05 and using the GraphPad Prism 6 procedure (GraphPad Software Inc., USA). The calcium source in diets was considered as the variation factor.

## 3. Results

### 3.1. Analyses of Ca and P Content in O. ficus-indica Cladodes, and the Soluble and Insoluble Fiber Extracted from O. ficus-indica

In order to formulate the experimental diets, a minerals analysis was performed. The calcium content of *O. ficus-indica* powder, soluble fiber and insoluble fiber was 10.5% ± 0.1%, 5.79% ± 0.04% and 4.73% ± 0.05%, respectively. The insoluble fiber extracted from *O. ficus-indica* cladodes had the lowest content of this mineral. Regarding phosphorus content, *O. ficus-indica* cladodes showed the highest values (0.17% ± 0.01%) compared with soluble fiber (0.10% ± 0.01%) and insoluble fiber (0.07% ± 0.01%). 

### 3.2. Chemical Composition and Mineral Content in Experimental Diets

To examine the chemical composition and mineral content in experimental diets, a proximate chemical analysis was realized. The results are shown in Table 2. No significant differences (*p* ≤ 0.05) were detected in moisture, lipid, protein, carbohydrate and ash contents between the experimental diets, which was reflected in a similar energy density of diets. Likewise, no significant differences in calcium content were detected (*p* ≤ 0.05) between diets. Regarding phosphorus content, our results showed that the control diet had a significantly (*p* ≤ 0.05) higher level of this mineral (0.33% ± 0.16%) compared with all experimental diets. On the other hand, potassium and magnesium contents were significantly higher (*p* ≤ 0.05) in the diet prepared with *O. ficus-indica* powder (0.46% ± 0.02% and 0.11% ± 0.05%, respectively). Finally, no statistically significant differences (*p* ≤ 0.05) were detected in the Ca/P molar ratio between experimental diets, whose values ranged from 1.79 to 1.89.

### 3.3. Serum Estrogen Levels in Ovariectomized Rats

**Figure 1:**
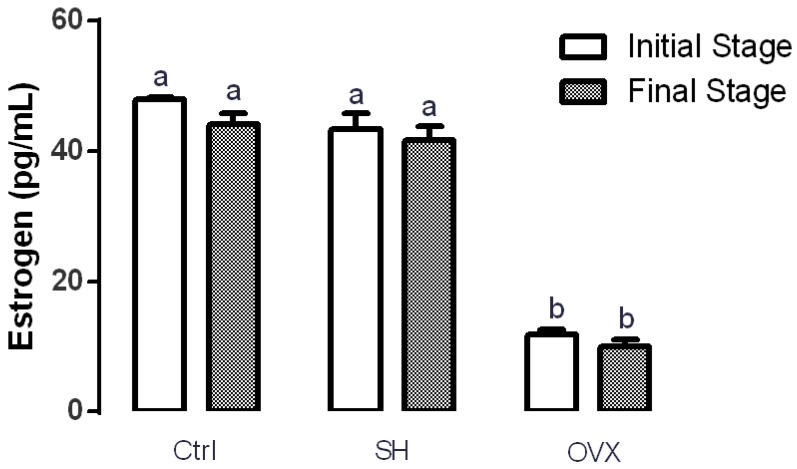
Effect of ovariectomy in serum estrogen levels at the beginning and at the end of the treatment. The results represent the average ± SD of the experimental groups, *n* = 5 in control (Ctrl), sham (SH) and ovariectomized (OVX) groups. Means in bars with different letters indicate significant differences (*p* ≤ 0.05) between groups with Tukey’s test.

Estrogen levels in rats were analyzed to ensure surgery success. Figure 1 shows that serum estrogen levels of rats from the control (47.97 ± 0.31 pg/mL) and sham (43.31 ± 0.28 pg/mL) groups at the beginning of the experiment were significantly higher (*p* ≤ 0.05) that those of the OVX group (11.91 ± 0.69 pg/mL). This same trend was observed at the end of the experiment. These results support the efficacy of ovariectomy for decreasing estrogen serum levels in rats.

### 3.4. Calcium Bioavailability

**Figure 2:**
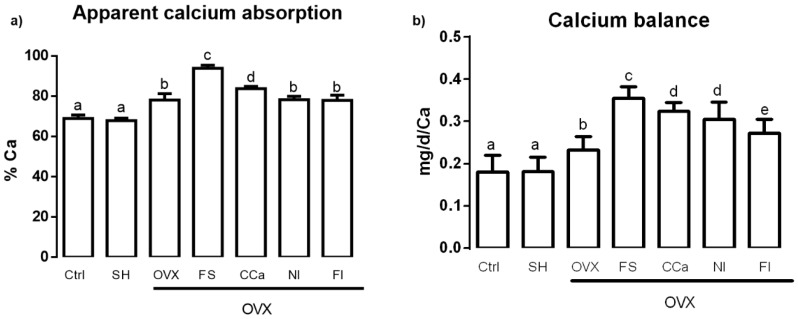
(**a**) Apparent calcium absorption and (**b**) calcium balance in experimental groups. The results represent the average ± SD of experimental groups, *n* = 5 rats/group. Means in bars with different letters indicate significant differences (*p* ≤ 0.05) between groups with Tukey’s test. Ctrl = control group, SH = sham group, OVX = ovariectomized group fed with AIN-93M, FS = ovariectomized group fed with diet supplemented with soluble fiber extracted from *O. ficus-indica*, CCa = ovariectomized group fed with diet supplemented with calcium citrate, NI = ovariectomized group fed with diet supplemented with *O. ficus-indica* powder, FI = ovariectomized group fed with diet supplemented with insoluble fiber extracted from *O. ficus-indica*.

Calcium bioavailability was studied by measuring the apparent calcium absorption and calcium balance. The results shown in Figure 2a indicate that the apparent calcium absorption was significantly higher (*p* ≤ 0.05) in the group fed with soluble fiber (93.87% ± 3.15%) compared with that observed in the groups fed with diets supplemented with calcium citrate (83.81% ± 2.12%), *O. ficus-indica* powder (78.26% ± 3.46%) and insoluble fiber (77.96% ± 5.16%) and the ovariectomized group (OVX) fed with AIN-93M (78.13% ± 6.31%). Rats that showed lower apparent calcium absorption belonged to the sham (67.89% ± 2.53%) and control (68.99% ± 3.35%) groups. Regarding calcium balance assessment (Figure 2b), we found that the highest value corresponded to the group fed with the diet supplemented with soluble fiber (0.35 ± 0.027 mg/d/Ca), which showed a significantly higher value (*p* ≤ 0.05) than the groups fed with diet supplemented with insoluble fiber (0.27 ± 0.03 mg/d/Ca), *O. ficus-indica* powder (0.30 ± 0.04 mg/d/Ca) and calcium citrate (0.32 ± 0.20 mg/d/Ca) and the OVX group fed with the standard diet (0.23 ± 0.03 mg/d/Ca). Finally, calcium balance in the control and sham groups was significantly lower (0.18 ± 0.04 mg/d/Ca and 0.18 ± 0.03 mg/d/Ca, respectively) compared with that of other experimental groups.

### 3.5. Physical and Biomechanical Properties of the Femur

To study the bone resistance to fracture, physical and biomechanical properties of femurs were analyzed. Femur length of rats from the OVX group (3.3 ± 0.25 cm) was significantly shorter (*p* ≤ 0.05) than that of the rest of the experimental groups as seen in Table 3. Furthermore, there were no statistical differences (*p* ≤ 0.05) in the weight, width and thickness of the femur of the animals corresponding to the different experimental groups. In the mechanical compression test (F_max_), the highest values were observed in the Ctrl (610.3 ± 46.6 N), SH (634 ± 45.8 N) and FS (606.5 ± 15.8 N) groups without significant statistical differences (*p* ≤ 0.05) between them. Contrastingly, a significantly lower value was detected in the OVX group (496.1 ± 24.9 N) in comparison with the rest of the experimental groups (*p* ≤ 0.05). In the three-point bending test (P_max_), no significant differences (*p* ≤ 0.05) were observed between the Ctrl (86.3 ± 5.5 N), SH (90.6 ± 1.1 N), FS (89.9 ± 4.3 N) and CCa (85.4 ± 4.4 N) groups. On the other hand, the OVX group displayed a significantly lesser P_max_ value (65.7 ± 3.5 N, *p* ≤ 0.05) compared with all the study groups. Regarding Young’s modulus (E), femurs of the rats fed with the diet supplemented with soluble fiber had significantly higher values (596.1 ± 78.4 N/mm^2^, *p* ≤ 0.05) than those shown by the rest of the groups, while the FI and OVX groups showed the lowest values (476.2 ± 19.7 and 439.3 ± 15.4 N/mm^2^, respectively).

### 3.6. Mineral Content in the Femur

Bone mineral absorption was determined with an analysis of mineral content in the target tissues (femurs). Table 4 shows the mineral content in the femur of experimental subjects. The highest calcium content was detected in the bones of SH (23.87% ± 0.19%), Ctrl (22.64% ± 0.20%), FS (22.91% ± 0.24%) and CCa (21.13% ± 0.18%) groups, while the groups that presented the lowest content of this mineral were the OVX (16.98% ± 0.98%), NI (20.41% ± 0.22%) and FI groups (20.11% ± 0.22%). Regarding the phosphorus content, there were no statistical differences (*p* ≤ 0.05) between the groups with the exception of the OVX group, which presented the lowest level of this mineral (5.18% ± 0.05%). Similarly, the femurs of rats in the OVX group showed the lowest content (0.14% ± 0.10%) of potassium with significant differences (*p* ≤ 0.05) between groups. In addition, the magnesium content in the bones of Ctrl and SH groups (0.74% ± 0.04% and 0.73% ± 0.02%, respectively) was significantly higher in comparison with the other experimental groups. Regarding the Ca/P ratio, the Ctrl (1.28 ± 0.06), SH (1.29 ± 0.01) and FS (1.23 ± 0.06) groups did not show statistical differences between them (*p* ≤ 0.05). However, the CCa, NI and FI groups showed significantly lower Ca/P ratio values (*p* ≤ 0.05) than the Ctrl, SH and SF groups. The highest value of the Ca/P ratio was detected in the OVX group (3.27 ± 0.16), which presented significant differences (*p* ≤ 0.05) compared with that detected in the rest of the experimental groups.

### 3.7. Bone Mineral Density (BMD) Measurements

**Figure 3:**
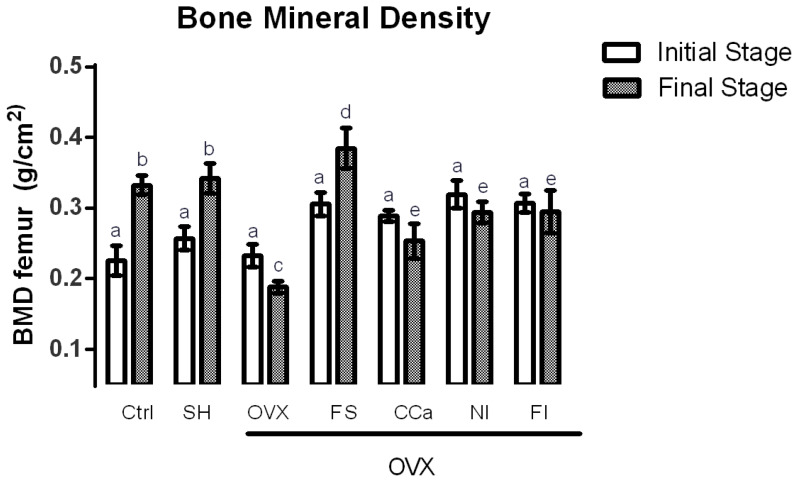
Bone mineral density in experimental groups. The results represent the average ± SD of experimental groups, *n* = 5 rats/group. Means in bars with different letters indicate significant differences (*p* ≤ 0.05) between groups with Tukey’s test. Ctrl = control group, SH = sham group, OVX = ovariectomized group fed with AIN-93M, FS = ovariectomized group fed with diet supplemented with soluble fiber extracted from *O. ficus-indica*, CCa = ovariectomized group fed with diet supplemented with calcium citrate, NI = ovariectomized group fed with diet supplemented with *O. ficus-indica* powder, FI = ovariectomized group fed with diet supplemented with insoluble fiber extracted from *O. ficus-indica*.

BMD measurements were performed to predict a decrease of bone mass as osteoporosis risk. The BMD in rats of experimental groups was recorded at the beginning and at the end of the experiment and the results are shown in Figure 3. Significant differences (*p* ≤ 0.05) in BMD were observed in rats from the CCa, NI, FI and OVX groups at the initial and final stages of the experiment. In general, BMD values of rats from these groups were lesser at the end of the treatment compared with the values registered at the beginning of the experiment. BMD measurements in the rats from the CCa, NI and FI groups at the final stage of the experiment were greater than those of the OVX group. By contrast, BMD values in rats from the Ctrl, SH and FS groups increased significantly (*p* ≤ 0.05). Interestingly, rats belonging to the FS group showed significantly higher BMD measurements (*p* ≤ 0.05) than rats from the Ctrl and SH groups.

### 3.8. Bone Microstructure Evaluation

**Figure 4:**
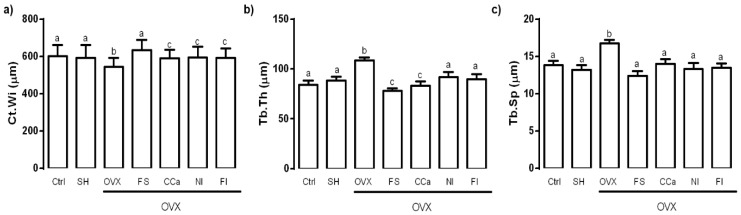
(**a**) Cortical thickness (Ct.Wi), (**b**) trabecular thickness (Tb.Th) and (**c**) trabecular separation (Tb.Sp) in femoral bone of experimental groups. The results represent the average ± SD of the experimental groups, *n* = 5 rats/group. Means in bars with different letters indicate significant differences (*p* ≤ 0.05) between groups with Tukey’s test. Ctrl = control group, SH = sham group, OVX = ovariectomized group fed with AIN-93M, FS = ovariectomized group fed with diet supplemented with soluble fiber extracted from *O. ficus-indica*, CCa = ovariectomized group fed with diet supplemented with calcium citrate, NI = ovariectomized group fed with diet supplemented with *O. ficus-indica* powder, FI = ovariectomized group fed with diet supplemented with insoluble fiber extracted from *O. ficus-indica*.

The femur microstructure was determined as it provides information regarding bone fragility. The cortical thickness (Ct.Wi) in femurs of experimental groups is shown in Figure 4a. No significant differences were observed between the Ct.Wi values obtained from femurs of rats of the Ctrl, SH and FS groups (*p* ≤ 0.05). However, the Ct.Wi values of these groups were significantly higher (*p* ≤ 0.05) than those of bones from rats of the CCa, FI and NI groups. The femoral Ct.Wi values in rats from the OVX group were significantly lower (*p* ≤ 0.05) in comparison with the rest of the experimental groups.

Regarding trabecular thickness (Tb.Th) in the femoral epiphysis, the OVX group showed a significantly higher value (*p* ≤ 0.05) compared with the other experimental groups. The lowest Tb.Th values were observed in bones from rats of the FS and CCa groups (Figure 4b). Trabecular separation (Tb.Sp) was significantly higher (*p* ≤ 0.05) in femurs from the OVX group compared to that observed in femurs from the other experimental groups (Figure 4c).

**Figure 5:**
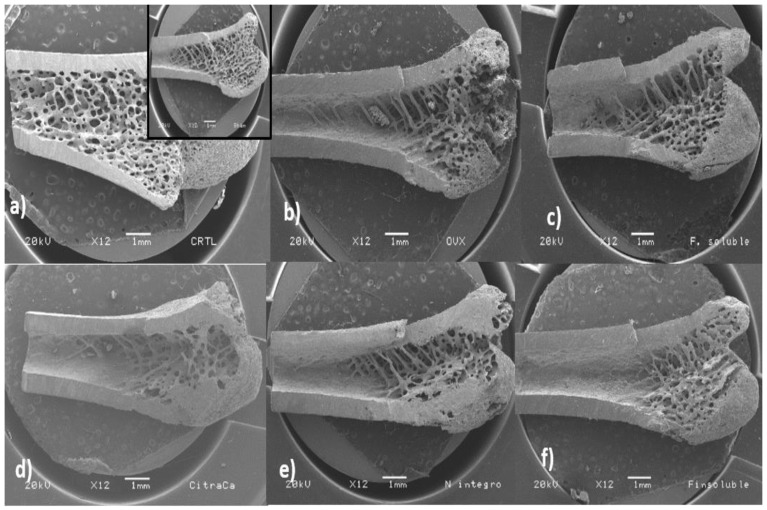
Scanning electron micrographs of the inner part of the femur (longitudinal cut from the line between condyles in the direction to the diaphysis at 12X) of rats from the experimental groups. (**a**) Ctrl and sham (insert in the upper right) groups, (**b**) ovariectomized group fed with AIN-93M, (**c**) ovariectomized group fed with diet supplemented with soluble fiber extracted from *O. ficus-indica*, (**d**) ovariectomized group fed with diet supplemented with calcium citrate, (**e**) ovariectomized group fed with diet supplemented with insoluble fiber extracted from *O. ficus-indica*, (**f**) ovariectomized group fed with diet supplemented with *O. ficus-indica* powder.

Scanning electron micrographs of the trabecular area of femurs from rats of the experimental groups are shown in Figure 5. It was evident that the trabecular tissue of femoral bones in the SH and Ctrl groups comprised an area that extended from the epiphysis to a third of the femur. Contrastingly, rats of the OVX group had trabecular areas that only covered the epiphysis. Furthermore, the metaphyseal cancellous tissue was reduced and disappeared completely in the diaphysis area. The trabecular area in femurs of rats from the NI and FS groups was very similar to that of bones of the OVX group. However, cancellous bone tissue from rats of the NI and FS groups was more homogeneous and the epiphyses looked more compact than those of rats from the OVX group. On the other hand, the areas corresponding to the trabecular tissue of rats from the CCa and IF groups were smaller compared with those of the rest of the experimental groups.

### 3.9. Determination of Biomarkers Related to Bone Remodeling

**Figure 6:**
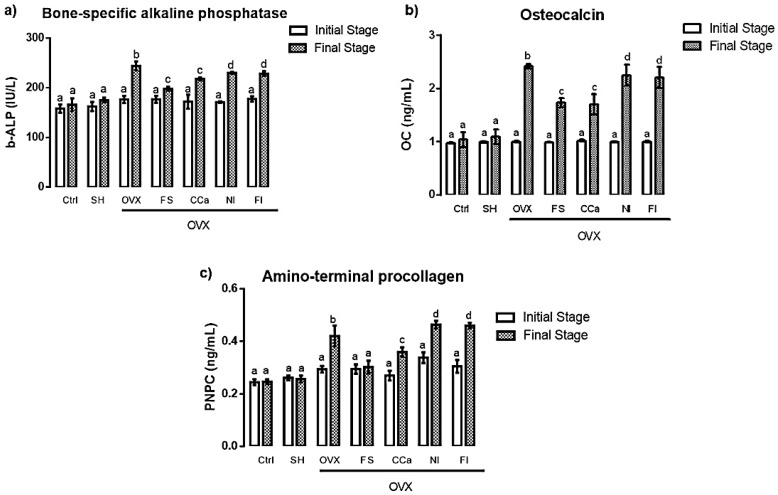
Serum levels of remodeling bone biomarkers at the initial and final stages of the treatments (**a**) alkaline phosphatase (b-ALP), (**b**) osteocalcin (OC) and (**c**) amino-terminal procollagen (PNPC). The results represent the average ± SD of experimental groups, *n* = 5 rats/group. Means in bars with different letters indicate significant differences (*p* ≤ 0.05) between groups with Tukey’s test. Ctrl = control group, SH = sham group, OVX = ovariectomized group fed with AIN-93M, FS = ovariectomized group fed with diet supplemented with soluble fiber extracted from *O. ficus-indica*, CCa = ovariectomized group fed with diet supplemented with calcium citrate, NI = ovariectomized group fed with diet supplemented with *O. ficus-indica* powder, FI = ovariectomized group fed with diet supplemented with insoluble fiber extracted from *O. ficus-indica*.

In order to evaluate bone metabolism in rats, the biomarkers, namely, bone-specific alkaline phosphatase, osteocalcin and amino-terminal procollagen, were analyzed. No significant difference (*p* ≤ 0.05) was found between initial and final rat serum values of alkaline phosphatase in the Ctrl and SH groups (Figure 6a). Contrastingly, levels of this remodeling biomarker were significantly higher at the end of the experiment in serum samples from rats of the OVX (244.27 ± 7.69 IU/L), NI (230.05 ± 4.33 IU/L) and FI (228.65 ± 6.32 IU/L) groups. Rats belonging to the CCa (218.02 ± 6.68 IU/L) and FS (198.21 ± 4.89 IU/L) groups exhibited intermediate values of this biomarker, which were significantly (*p* ≤ 0.05) different from those of the control (166.13 ± 5.35 IU/L) and SH (175.40 ± 2.92 IU/L) groups.

Figure 6b shows osteocalcin serum levels. At the beginning of the experiment, no significant differences (*p* ≤ 0.05) were detected between the groups. However, at the end of the treatments, significant differences (*p* ≤ 0.05) in the serum levels of this biomarker were observed between the experimental groups. The same trend, as that observed for alkaline phosphatase, was found for osteocalcin serum levels, where the highest value corresponded to rats of the OVX group (2.41 ± 0.09 ng/mL), followed by the NI (2.24 ± 0.44 ng/mL) and FI (2.20 ± 0.43 ng/mL) groups. The lowest levels of this biomarker were detected in the Ctrl (1.03 ± 0.27 ng/mL) and SH (1.09 ± 0.30 ng/mL) groups. Intermediate levels of osteocalcin were found in the CCa (1.70 ± 0.43 ng/mL) and SF (1.72 ± 0.19 ng/mL) groups.

Finally, amino-terminal procollagen (PNPC) serum levels are shown in Figure 6c. No significant differences (*p* ≤ 0.05) were found between the groups at the beginning of the experiment. Nevertheless, at the end of the treatments, the highest serum levels of this biomarker belonged to rats from the NI (0.46 ± 0.01 ng/mL) and FI (0.47 ± 0.01 ng/mL) groups, which showed significant differences (*p* ≤ 0.05) compared with those of rats from the OVX group, which displayed high levels of this biomarker (0.42 ± 0.04 ng/mL). Intermediate serum levels corresponded to rats of the CCa group (0.35 ± 0.01 ng/mL), while the lowest levels were detected in rats from the Ctrl (0.24 ± 0.09 ng/mL), SH (0.25 ± 0.01 ng/mL) and FS (0.30 ± 0.01 ng/mL) groups.

## 4. Discussion

The consumption of foods rich in calcium is recommended to prevent bone diseases. Particularly, postmenopausal women have an increased risk of suffering from this disease due to the hormonal decrease of estrogens [21]. One of the most commonly used experimental models to study this condition is the ovariectomized rat model of postmenopausal bone loss [22], which was employed in the present study. As expected, the experimental group that underwent ovariectomy showed significantly lesser serum estrogen levels compared with the simulated surgery group (SH) and the control group (Ctrl). Reported values of circulating estrogen in healthy 3-month-old female Wistar rats range from 40 to 55 pg/mL, while ovariectomized rats of the same age show estrogen levels ranging from 5 to 12 pg/mL [23,24,25]. In this study, estrogen levels of rats from the Ctrl (47.9 ± 0.3 pg/mL) and SH (43.3 ± 2.5 pg/mL) groups were within the range previously reported in healthy rats. Estradiol serum levels of ovariectomized rats (11.2 ± 0.6 pg/mL) were also similar to those reported in previous studies [25].

The dietary fiber improves mineral absorption and therefore, favors mineral bioavailability [26]. Soluble fiber contains inulin, which is not digested during its passage through the intestine and therefore, remains intact until it reaches the colon, where it is fermented by the microbiota, which stimulate the growth of *Bifidobacterium* strains, subsequently modifying intestinal pH [27]. Soluble fiber fermentation produces short-chain fatty acids, such as butyrate, propionate and acetate, among other organic acids [28]. These fatty acids diminish the luminal pH in the colon and, as a consequence, Ca^2+^ absorption is augmented by a mechanism that comprises the exchange of cellular H^+^ for luminal Ca^2+^. In addition, absorption of this mineral by passive diffusion is also increased [29].

On the other hand, CO_2_ (another product of fiber fermentation), in the presence of bicarbonate, which is secreted by the pancreas in order to neutralize the acidity achieved by fatty acids [30], interacts with calcium carbonate, which is found in soluble fiber [10]. This reaction releases ionic calcium and contributes to maintain an optimal pressure of intestinal CO_2_, which in turn improves the absorption of this mineral [31]. In vitro studies have demonstrated that CO_2_ production helps to solubilize calcium, when it is in the form of carbonate [32]. In fact, the administration of calcium carbonate (1000 mg/day) to premenopausal women increased serum calcium levels and reduced circulating levels of parathyroid hormone more efficiently than the administration of calcium citrate (1000 mg/day) [33].

The results obtained from the determination of the apparent absorption of calcium correlate with the reabsorption of calcium in the renal tubules. Approximately, 20% of filtered calcium is reabsorbed into the loop of Henle by means of the Na^+^/K^+^ pump and Ca^2+^/Na^+^ exchanger. In the distal convoluted tubule, approximately 8% of the filtered calcium is actively reabsorbed; therefore, this is the segment where the highest regulation of calcium excretion occurs [34]. Rats from the FS group had the highest value of calcium balance (0.35 mg/d/Ca), which indicated a better efficiency of calcium reabsorption compared with that of rats from the CCa, NI and IF groups. It is worth mentioning that diets administered to rats of the FI and NI groups contained higher levels of insoluble fiber, which is the least fermentable fiber in the colon [28]. Insoluble fiber is rich in cellulose, which limits the accessibility of fermentation enzymes in the colon, producing only acetic acid [35]. On the contrary, final products of soluble fiber fermentation (short-chain fatty acids, lactic acid, etc.) are more numerous and varied, which allow better calcium reabsorption [30].

Ovariectomized rats (OVX group) showed lower calcium balance values than rats from the FS, FI, CCa and NI groups. This can be attributed to the lack of estrogens, which provoke an increased calcium loss through bone resorption and hypercalciuria [36]. The main regulator of renal calcium excretion is parathyroid hormone (PTH) and its secretion can be indirectly activated by growth factor 23 (FGF23), which is synthesized in the osteoblasts and interacts with FGF23 receptors that require a Kloto co-receptor (whose gene is expressed predominantly in kidney) [37]. It has been demonstrated that estrogens increase FGF23 mRNA expression, so that in the absence of estradiol, this expression diminishes, causing a rise in glomerular filtration rate and a decrease in tubular resorption, which lead to calciuria [38]. In the case of rats from the Ctrl and SH groups, a net calcium absorption and an adequate calcium balance were observed. Contrastingly, ovariectomized rats, whose calcium absorption was higher than that of rats from the Ctrl and SH groups, experienced a higher calcium loss, as evidenced by their calcium balance values. 

Regarding the analysis of the physical and biomechanical properties of the rat femoral bones, it was evident that rats from the Ctrl, SH and FS groups showed the highest values in the compression and three-point bending tests, which indicated that greater force was required to achieve bone breakage. These results are according to Hernández-Becerra et al. [9], who reported that bones of rats in growing stage, fed with *O. ficus-indica* cladodes at a late maturity stage (100 and 135 days) as the only source of calcium, required greater strength for bone fracture than the bones of rats fed with cladodes at an early maturity stage (25 and 60 days).

Probably, a lower mechanical resistance in the bones of rats from the NI and FI groups was due to the presence of calcium oxalate and phytates in *O. ficus-indica* cladodes at a late maturity stage and their insoluble fiber. These components act as chelating agents of various minerals, such as calcium, magnesium, copper and zinc, resulting in a lower absorption of these minerals, as reported in previous studies regarding the presence of mineral-sequestering agents in *O. ficus-indica* species and their influence on calcium bioavailability [39]. 

Femurs from rats fed with diets supplemented with calcium carbonate (CCa group) showed less mechanical resistance and apparent calcium absorption than bones of rats fed with diets supplemented with *O. ficus-indica* soluble fiber. The solubility product constant (K_sp_) value of calcium carbonate is lower (3.36 × 10^−9^) than that of calcium citrate (9.6 × 10^−2^). However, it is important to take into account that these values are modified depending on the pH. In the case of the gastrointestinal tract, pH values greatly vary from highly acid in the stomach to a pH value of 6 (approximately) in the duodenum; therefore, the solubility of the compounds that transit in the intestine changes. That is the case for calcium salts, whose absorption and bioavailability differs [33], as has been demonstrated in a clinical study carried out in adult women (>45 years old), who ingested calcium carbonate, calcium lactate, calcium glutamate and calcium citrate as sources of calcium. The investigation indicated that the absorption and bioavailability of calcium lactate was significantly higher than that of the other three calcium salts. The absorption and bioavailability of calcium carbonate was better than that of calcium citrate, whereas calcium glutamate had the lowest values [40]. 

Regarding the elasticity bone module test (Young’s module test), the highest values corresponded to femurs of rats of the FS group. This means that these bones will undergo small deformations with great efforts [41]. 

On the other hand, calcium content of femoral rat bones from the Ctrl, SH, FS and CCa groups did not show statistical differences (*p* ≤ 0.05), while bones of rats from the OVX group presented lower calcium content. Hydroxyapatite (Ca_5_(PO_4_)_3_(OH)) is the main bone component, which confers rigidity and resistance to bones [42]. Therefore, calcium and phosphorus are essential for bone formation (mineralization), although Mg, K and Zn (trace elements) are also bone components [43].

The Ca/P ratio in bone has been used as an indicator for osteoporosis. Previous studies have indicated that estrogen deficiency induced by ovariectomy in female rats leads to an increase in Ca/P ratios in both tibial and femoral bones. The Ca/P ratio observed in ovariectomized rats is >2.0, whereas healthy female rats show values <2.0 [44,45,46]. Ca/P ratio increment in rats that underwent ovariectomy suggests a negative effect on the balance between bone resorption and bone formation activity [43]. In this study, the Ca/P ratio was <2.0 in rats of the control and sham groups and in the ovariectomized rats which were fed with diets containing calcium citrate, *O. ficus-indica* powder and soluble and insoluble fiber from *O. ficus-indica*. However, the Ca/P ratio was lower in bones from rats of the CaC, NI and FI groups, which indicated inadequate calcium absorption reflected in a decrease in bone mineralization [47]. 

A satisfactory calcium intake accompanied by adequate bioavailability increases BMD [33]. BMD values diminish when estrogen production is reduced due to the constant loss of calcium, provoked by an increment in bone resorption [48]. Wistar rats with ovariectomy show femoral mean BMD values of 0.208 g/cm^2^ [49], In this investigation, rats that showed the highest BMD measurements were those fed with a diet containing the *O. ficus-indica* soluble fiber. It has been previously reported that mucilage from *O. ficus-indica* is constituted of soluble dietary fiber (ranging from 51.70% to 67.51%) [50]. This polysaccharide consists of alternating rhamnose and galacturonic acid residues, which are attached to the side chains composed of three galactose residues. In addition, arabinose and xylose sugars are branched on the galactose side chains [51]. These structural characteristics of soluble fiber from *O. ficus-indica* positions it as a potential prebiotic, that can be hydrolyzed and fermented by the colonic microbiota in the large intestine [26]. 

Prebiotic fibers have been associated with increases in bifidobacteria in doses up to 20 g/day, administered for 7 to 64 days in human adults [52]. It has been observed that the type of prebiotic fiber favors the production of bifidobacteria and contributes to a better mineral absorption, which is reflected in a higher BMD [53]. Numerous studies have repeatedly shown that prebiotics, such as oligofructose, inulin and galacto-oligosaccharides, effectively stimulate calcium absorption in rats [29,54,55,56,57,58]. This fact was previously observed, where loss of minerals in bones of menopausal women and ovariectomized rats was prevented with a combination of prebiotics (oligofructose plus *Acacia* gum) added to experimental diets [59,60].

To complement bone quality characterization, the microstructure of the rat femoral bones was analyzed. The trabecular and compact bone act together in order to meet the physiological needs of the organism [42]. The proportion of trabecular and cortical bone can affect fracture resistance, while the negative imbalance of remodeling and increased bone resorption cause greater porosity in both trabecular and cortical areas, which are factors to increase the risk of fracture [61]. In this study, femurs of rats from the OVX group had a lower cortical thickness, which consequently had a negative impact on the mechanical tests, since femoral bones of rats belonging to this group displayed a lower resistance to fracture. By contrast, femurs of rats from the Ctrl, SH and FS groups showed a greater resistance to bone breakage. This was in accordance with their greater cortical thickness (Ct.Wi) and less trabecular space (Tb.Sp).

Interestingly, Tb.Th values observed in femoral bones of rats from the FS group were similar to those of rats from the CCa group, indicating that the effect of calcium present in the soluble fiber extracted from *O. ficus inica* might be comparable to that of calcium citrate, a common supplement recommended in patients with osteoporosis [62]. Micrographs of femoral bones of rats from the FS group showed that the trabecular zone covered part of the diaphysis, unlike what happened in bones of rats from the CCa group, in which the trabecular zone was only observed in the metaphysis. In the case of femoral bones of rats from the Ctrl and SH groups, the trabecular zone completely covered the diaphysis, confirming that these experimental subjects did not experience calcium loss due to estrogen absence. Regarding rats fed with diets containing *O. ficus-indica* powder and its insoluble fiber, it was evident that they had a significant bone resorption, since the trabecular area was restricted to a part of the femoral epiphysis.

Bones of ovariectomized rats had the greatest values of trabecular thickness. Moreover, some fissures were observed in their bones, indicating a destruction of bone tissue, which evidently was related to low calcium content. Our results are in accordance with previous studies, which demonstrated that ovariectomized rats had low BMD values and their bones had large trabecular thickness and lower resistance to fracture, which was attributed to the presence of fissures and a greater porosity of the bones [47,60]. Bones of rats of the other experimental groups did not show significant differences in terms of their trabecular separation. 

Rats from the CCa, IF and NI groups had a higher percentage of calcium and phosphorus than rats of the OVX group, as well as greater absorption and calcium balance. These findings were indicative of osteoblastic activation to recover bone loss provoked by osteoclastic action. However, recovery of bones of these rats was not completely achieved (Figure 5). On the other hand, greater trabecular areas were observed in femoral bones of rats fed with diets containing soluble fiber extracted from *O. ficus-indica*, indicating that this component acts a protector for osteoblastic action.

Bone-specific alkaline phosphatase, amino-terminal procollagen and osteocalcin are the most commonly biomarkers used to study bone remodeling. Mineralization is the formation of hydroxyapatite crystals in the superficial membrane of the osteoblasts, followed by the propagation of hydroxyapatite in the extracellular matrix and its deposition between the collagen fibrils [63]. This occurs through extracellular inorganic pyrophosphate that is provided by nucleotide pyrophosphatase phosphodiesterase 1, so the role of the alkaline phosphatase is to hydrolyze pyrophosphate and provide inorganic phosphate to promote bone mineralization [64]. High levels of alkaline phosphatase have been observed in both postmenopausal women and ovariectomized rats, due to the increase in bone remodeling [43]. Reported serum level of bone-specific alkaline phosphatase in healthy adult Wistar rats is <200 IU/L, while in ovariectomized rats the level is >200 IU/L [65]. In this study, rats that underwent ovariectomy showed high values of this marker (>200 IU/L) at the end of the experiment, indicating an increase in bone remodeling due to the negative balance in bone resorption. In contrast, rats from the FS group showed values <200 IU/L, which might reflect a protective mechanism against osteoclastic resorption due to the effectiveness of calcium absorption promoted by the soluble fiber of *Opuntia ficus-indica* in diet. Osteocalcin is a peptide synthesized by osteoblasts in the last stages of bone formation in conjunction with vitamin D [66]. The levels of this biochemical marker of bone remodeling, both in postmenopausal women and in adult ovariectomized Wistar rats, are high. Reference values in healthy adult rats are <1.0 ng/mL and in ovariectomized rats are >1.0 ng/mL [67,68]. In the case of rats from the OVX, NI and FI groups, osteocalcin serum values were above 2.0 ng/mL. These results further confirmed that rats had experienced bone resorption, and therefore, it was necessary to activate osteoblasts to initiate bone remodeling in such a way that bone mineralization is “increased”, in order to recover bone lost by the action of osteoclasts. On the contrary, Ctrl, SH, FS and CCa groups showed values below 2.0 ng/mL, indicating that a marked osteoblastic activation was not necessary as in the case of rats from the OVX, NI and FI groups.

Collagen is a heterodimer that contributes to the integrity and strength of bone matrix. Serum values of this marker increase during growth and in situations of augmented bone formation [69]. Reported collagen levels in healthy Wistar rats are <0.20 mg/L, while those of ovariectomized Wistar rats are >0.20 mg/L [48,70]. In the present study, serum levels of this biomarker displayed the same trend as that of alkaline phosphatase and osteocalcin; rats from the OVX, NI and FI groups showed values >0.40 mg/L, while rats from the FS group showed values <0.40 mg/L. 

In summary, serum levels of biomarkers related to bone remodeling in rats fed with diets containing *O. ficus-indica* powder, insoluble fiber and calcium citrate were similar to those of rats with estrogen deficiency and weakened bones. However, these biomarker values in rats fed with the soluble fiber of *O. ficus-indica* were consistent with improved calcium absorption, which avoids the need for increased remodeling activity. 

The present study demonstrated the beneficial effects of the consumption of soluble fiber from *O. ficus-indica* on the physicochemical and structural properties of bones. Nevertheless, the short- and long-term effects of low, medium and high doses of the soluble fiber were not assessed in order to determine the minimum effective dose required to improve bone structure and strength. Evidently, it is also necessary to establish if soluble fiber extracted from *O. ficus-indica* is able to favor calcium absorption and induce changes in gut microbial bacteria, so it could be considered as a prebiotic. It is also important to further assess the influence of this soluble fiber on osteoblastic/osteoclastic activity balance and to analyze the percentage of calcium from *O. ficus-indica*’s soluble fiber that is fixed directly to the bone. These analyses are worthy of further study and will allow a broader understanding of the specific mechanisms by which the soluble fiber of *O. ficus-indica* contributes to preserve bone health.

## 5. Conclusions

The highest values of apparent calcium bioavailability, calcium absorption, fracture resistance, calcium content and bone mineral density were obtained in rats, whose diet was supplemented with the soluble fiber extracted from *O. ficus-indica*. Moreover, femurs from these rats had greater cortical thickness and trabecular separation, which evidently contributed to improve their bone mineral density and biomechanical properties, as clearly evidenced by a better resistance to fracture. Serum values of bone remodeling biomarkers were lower in rats fed with the diet containing the soluble fiber of *O. ficus-indica*. Our results demonstrated that consumption of the soluble fiber extracted from *O. ficus-indica* cladodes protects bones from osteoclastic activity, therefore contributing to improve the physical, densitometric, biomechanical and microstructural properties of bones. Further work is needed to identify the exact mechanisms involved by which soluble fiber extracted from *O. ficus-indica* improves calcium absorption and the role of this soluble fiber as a prebiotic and to evaluate different concentrations of soluble fiber in the diet to identify the adequate dose that improves calcium bioavailability, when the level of this mineral in the diet is low.

## Figures and Tables

**Table 1:** Ingredient composition of the experimental diets (g/kg).

	Groups	Control (Ctrl)	Calcium Citrate (CCa)	*O. ficus-indica* Powder (NI)	Soluble Dietary Fiber (FS)	Insoluble Dietary Fiber (FI)
Ingredients						
Corn starch		621	621	621	621	621
Sucrose		140	140	140	140	140
Casein ^a^		100	100	100	100	100
Soybean oil		40	40	40	40	40
Fiber ^b^		50	50	33	43	35
MixMin ^c^		10	10	10	10	10
MixVit ^d^		35	35	35	35	35
L-Cystine		1.8	1.8	1.8	1.8	1.8
Choline bitartrate		2.5	2.5	2.5	2.5	2.5
CaCO_3_^e^		12.5	-	-	-	-
Calcium citrate		-	0.207	-	-	-
*O. ficus-indica* powder		-	-	48	-	-
Soluble fiber extracted from *O. ficus-indica*		-	-	-	86	-
Insoluble fiber extracted from *O. ficus-indica*		-	-	-	-	105

^a^ C-7078, Casein Sigma Chemical, Inc., St. Louis, MO, USA. ^b^ α-Cell Fiber Solft Zolca, MP Biomedicals, Santa Ana, CA, USA. ^c^ Mineral mix without calcium (AIN-93-MX, Harlan Inc., IN, USA, TD 04374) [13]. ^d^ Vitamin mix (AIN-93-VX, Harlan Inc., IN, USA, TD 94047) [13]. ^e^ Control diet contained CaCO_3_ (Merck 2066, Darmstadt, Germany) as the calcium source. In experimental diets, *O. ficus-indica* powder provided 5 g/kg of calcium, as well as carbohydrates, proteins and lipids, that complemented nutritional requirements in experimental diets (AIN-93G) [13].

**Table 2:** Proximate chemical composition, mineral content and Ca/P ratio of control and experimental diets (g/100 g).

Content	Ctrl	CCa	NI	FS	FI
Moisture	4.50 ± 0.02 ^a^	4.38 ± 0.02 ^a^	4.49 ± 0.02 ^a^	4.45 ± 0.03 ^a^	5.00 ± 0.06 ^a^
Ashes	3.24 ± 0.01 ^a^	3.28 ± 0.02 ^a^	3.60 ± 0.08 ^a^	3.19 ± 0.03 ^a^	3.37 ± 0.01 ^a^
Carbohydrates **	76.90 ± 7.69 ^a^	73.98 ± 7.39 ^a^	80.65 ± 8.0 ^a^	74.92 ± 7.49 ^a^	77.70 ± 7.77 ^a^
Protein **	11.94 ± 0.10 ^a^	13.30 ± 0.91 ^a^	11.58 ± 0.30 ^a^	11.68 ± 1.03 ^a^	11.54 ± 0.10 ^a^
Lipids **	3.43 ± 0.37 ^a^	3.06 ± 0.10 ^a^	3.68 ± 0.11 ^a^	3.76 ± 0.16 ^a^	3.39 ± 0.14 ^a^
cal/kg diet *	386.21 ± 0.16 ^a^	394.67 ± 0.17 ^a^	376.02 ± 0.15 ^a^	371.21 ± 0.15 ^a^	378.42 ± 0.18 ^a^
Magnesium	0.06 ± 0.01 ^a^	0.05 ± 0.01 ^a^	0.11 ± 0.02 ^b^	0.08 ± 0.01 ^c^	0.09 ± 0.01 ^c^
Potassium	0.29 ± 0.01 ^a^	0.20 ± 0.01 ^a^	0.46 ± 0.02 ^b^	0.33 ± 0.01 ^c^	0.34 ± 0.02 ^c^
Calcium	0.59 ± 0.03 ^a^	0.54 ± 0.02 ^a^	0.55 ± 0.03 ^a^	0.55 ± 0.06 ^a^	0.54 ± 0.03 ^a^
Phosphorus	0.33 ± 0.02 ^a^	0.28 ± 0.01 ^b^	0.29 ± 0.01 ^b^	0.29 ± 0.02 ^b^	0.29 ± 0.01 ^b^
Ca/P ratio	1.79 ± 0.12 ^a^	1.89 ± 0.07 ^a^	1.86 ± 0.10 ^a^	1.87 ± 0.20 ^a^	1.83 ± 0.11 ^a^

* Caloric percentage contribution of carbohydrates, proteins and lipids in diets. ** Energy values of carbohydrates, proteins and lipids were 4, 4 and 9 kcal/g, respectively. The values represent mean ± standard deviation (SD), *n* = 5 rats/group. Means in rows with different letters differ significantly (*p* ≤ 0.05).

**Table 3:** Physical and mechanical properties of femoral bone in rats fed with the experimental diets.

Parameters	Ctrl	SH	OVX	FS	CCa	NI	FI
Length (cm)	3.6 ± 0.16 ^a^	3.5 ± 0.15 ^a^	3.3 ± 0.25 ^b^	3.6 ± 0.12 ^a^	3.6 ± 0.08 ^a^	3.6 ± 0.13 ^a^	3.6 ± 0.13 ^a^
Weight (g)	0.92 ± 0.16 ^a^	0.94 ± 0.03 ^a^	0.97 ± 0.09 ^a^	0.95 ± 0.12 ^a^	0.96 ± 0.07 ^a^	0.97 ± 0.05 ^a^	0.94 ± 0.08 ^a^
Width (mm)	4.0 ± 0.10 ^a^	4.0 ± 0.09 ^a^	4.0 ± 0.10 ^a^	4.0 ± 0.11 ^a^	4.0 ± 0.15 ^a^	4.0 ± 0.10 ^a^	4.0 ± 0.10 ^a^
Thickness (mm)	5.1 ± 0.05 ^a^	5.1 ± 0.03 ^a^	5.0 ± 0.05 ^a^	5.1 ± 0.04 ^a^	5.2 ± 0.06 ^a^	5.1 ± 0.05 ^a^	5.1 ± 0.05 ^a^
Compression test F_max_ (N)	610.3 ± 46.6 ^a^	634 ± 45.8 ^a^	496.1 ± 24.9 ^b^	606.5 ± 15.8 ^a^	555.9 ± 14.8 ^c^	530.9 ± 37.7 ^c^	533.4 ± 26.5 ^c^
Three-point bending test P_max_ (N)	86.3 ± 5.5 ^a^	90.6 ± 1.1 ^a^	65.7 ± 3.5 ^b^	89.9 ± 4.3 ^a^	85.4 ± 4.4 ^a^	74.87 ± 3.6 ^c^	73.0 ± 5.9 ^c^
*E* (N/mm^2^)	545.3 ± 82.5 ^a^	556.1 ± 23.7 ^a^	439.3 ± 15.4 ^b^	596.1 ± 78.4 ^c^	556.6 ± 19.6 ^a^	539.3 ± 15.9 ^a^	476.2 ± 19.7 ^b^

The values represent mean ± SD, *n* = 5 rats/group. Means in rows with different letters differ significantly (*p* ≤ 0.05). F_max_: failure load evaluated by the compression test, P_max_: failure load evaluated by the three-point bending test. Ctrl = control group, SH = sham group, OVX = ovariectomized group fed with AIN-93M, FS = ovariectomized group fed with diet supplemented with soluble fiber extracted from *O. ficus-indica*, CCa = ovariectomized group fed with diet supplemented with calcium citrate, NI = ovariectomized group fed with diet supplemented with *O. ficus-indica* powder, FI = ovariectomized group fed with diet supplemented with insoluble fiber extracted from *O. ficus-indica*.

**Table 4:** Mineral content in femoral bone of rats fed with the experimental diets.

Group	Ca (%)	P (%)	K (%)	Mg (%)	Ca/P Ratio
**Ctrl**	22.64 ± 0.20 ^a^	17.66 ± 0.13 ^a^	0.21 ± 0.04 ^a^	0.74 ± 0.04 ^a^	1.28 ± 0.06 ^a^
**SH**	23.87 ± 0.19 ^a^	18.46 ± 0.14 ^a^	0.21 ± 0.06 ^a^	0.73 ± 0.02 ^a^	1.29 ± 0.01 ^a^
**OVX**	16.98 ± 0.98 ^b^	5.18 ± 0.05 ^b^	0.14 ± 0.10 ^b^	0.51 ± 0.02 ^b^	3.27 ± 0.16 ^b^
**FS**	22.91 ± 0.24 ^a^	18.60 ± 0.12 ^a^	0.19 ± 0.04 ^a^	0.43 ± 0.02 ^b^	1.23 ± 0.06 ^a^
**CCa**	21.13 ± 0.18 ^a^	17.99 ± 0.14 ^a^	0.18 ± 0.09 ^a^	0.44 ± 0.02 ^b^	1.17 ± 0.05 ^c^
**NI**	20.41 ± 0.22 ^c^	18.32 ± 0.09 ^a^	0.20 ± 0.04 ^a^	0.47 ± 0.02 ^b^	1.11 ± 0.08 ^c^
**FI**	20.11 ± 0.22 ^c^	18.77 ± 0.15 ^a^	0.19 ± 0.04 ^a^	0.45 ± 0.02 ^b^	1.07 ± 0.05 ^c^

The values represent mean ± SD, *n* = 5 rats/group. Means in columns with different letters differ significantly (*p* ≤ 0.05). Ctrl = control group, SH = sham group, OVX = ovariectomized group fed with AIN-93M, FS = ovariectomized group fed with diet supplemented with soluble fiber extracted from *O. ficus-indica*, CCa = ovariectomized group fed with diet supplemented with calcium citrate, NI = ovariectomized group fed with diet supplemented with *O. ficus-indica* powder, FI = ovariectomized group fed with diet supplemented with insoluble fiber extracted from *O. ficus-indica*.
